# A Matter of Feelings: Mediators’ Perceptions of Emotion in Hierarchical Workplace Conflicts

**DOI:** 10.3389/fpsyg.2021.629768

**Published:** 2021-06-03

**Authors:** Meriem Kalter, Katalien Bollen, Martin Euwema, Alain-Laurent Verbeke

**Affiliations:** ^1^Department of Occupational and Organizational Psychology and Professional Learning, KU Leuven, Leuven, Belgium; ^2^Centre for Social Innovation (KSI), HU University of Applied Sciences Utrecht, Utrecht, Netherlands; ^3^Faculty of Law, KU Leuven, Leuven, Belgium

**Keywords:** emotions, emotion recognition, hierarchical labor conflict, mediator perception, workplace mediation

## Abstract

Emotions play a central role in the process of conflict and resolution. For a mediator, it is important to recognize emotions correctly and act upon them. Whether interventions are appropriate depends to a large extent on the ability of mediators to accurately perceive the emotions of conflict parties. Particularly in hierarchical labor conflicts, this can be challenging, since subordinates tend to hide emotions while supervisors tend to express them. In this study, we investigated if subordinates and supervisors differ in their emotional experience during mediation and whether mediators perceive these emotions accurately. To this end, we compared the extent to which disputants experienced certain emotions with the extent to which mediators perceived these emotions. Data were collected through surveys of mediation clients and mediators in hierarchical labor conflicts in the Netherlands. As expected, subordinates experienced a higher level of negative emotions during the mediation than supervisors did. Positive emotions, however, were experienced to a similar extent by both supervisors and subordinates in mediation. Mediators perceived supervisors’ emotions more accurately than they did subordinates’ emotions. While supervisors’ emotions were positively related with mediators’ perceptions, this was not the case for subordinates’ emotions. Furthermore, mediators were more accurately perceiving supervisors’ negative emotions than their positive emotions. Implications for mediation theory and practice are discussed.

## Introduction

Conflicting parties often experience emotions during mediation. One of the factors contributing to a successful mediation is that mediators acknowledge these emotions and set up a process to manage them (Jones and Bodtker, 2001; Bollen and Euwema, 2014; Ladd and Blanchfield, 2016). This could pave the way for conflict transformation and parties’ positive evaluation of the mediation (Jameson et al., 2009, 2014). In order to effectively handle emotions, mediators need to be accurate in perceiving them. That is, mediators perceive emotions as closely as possible as experienced by the parties themselves. The less accurate, the more they might intervene ineffectively, not matching parties’ emotional needs. However, are mediators able to achieve this? Several studies show that people have difficulties to accurately noting and “reading” emotions, though some are more “emotionally intelligent” than others (Kelly and Kaminskienë, 2016; Remland, 2016; Skordoulis et al., 2020). Mediators may be no exception to this (Jones and Bodtker, 2001; Smilovitz and Capelos, 2008; Charkoudian et al., 2009; Jameson et al., 2010; Zariski, 2010). Particularly in the context of hierarchical labor conflicts, emotion recognition can be challenging for mediators since subordinates tend to hide even strong emotions, while supervisors tend to express emotions, however small or limited they may be (Galinsky et al., 2008; Bombari et al., 2017). This can lead to misinterpretation of parties’ emotions by the mediator. For example, if mediators are better at recognizing supervisors’ emotions because these are more easily expressed, mediators may develop interventions that are more aligned with the supervisors’ needs and wishes. This can be particularly problematic since research shows that emotion acknowledgment by mediators is more important for subordinates to perceive the mediation as effective than it is for supervisors (Bollen and Euwema, 2014).

While many studies have explored the role of emotion in conflict (e.g., Nair, 2008; Lindner, 2014; Friedman et al., 2018) and negotiation (e.g., Adler et al., 1998; Druckman and Olekalns, 2008; Van Kleef and De Dreu, 2010), little empirical research has considered the role of emotion in mediation (Jones and Bodtker, 2001; Jameson et al., 2009). To examine whether mediators have an accurate perception of the emotions of their clients, it is necessary to first explore what emotions parties experience during mediation and whether they differ for parties who are in a different formal power position. The aim of the present study is therefore twofold. Firstly, it examines emotions of supervisors and subordinates in the mediation. Secondly, it explores mediators’ abilities to recognize them.

To date, most studies on emotions in mediation have focused on the importance of addressing negative emotions (Jones and Bodtker, 2001; Jones, 2006; Jameson et al., 2010; Picard and Siltanen, 2013), with few investigating disputants’ broader experience of emotions in mediation or whether mediators are able to accurately perceive these emotions. In addition, most studies are limited to negative emotions, such as anger (Fitness, 2000; Bollen and Euwema, 2014; Williams and Hinshaw, 2018). Therefore, this study investigates positive emotions, including happiness and enthusiasm, and a broader spectrum of negative emotions, including anger, fear and sadness.

Previous researchers differentiate between expressing and experiencing emotions since people sometimes do not show the emotions they are feeling or may fake an emotional reaction (Davis, 1995; Gibson and Callister, 2010; Vuori et al., 2018). For example, a subordinate might hide her anger because she is afraid of repercussions. For mediators, it is important to address expressed emotions, genuine or not, because of their impact on the conflict and the mediation process (Poitras and Raines, 2013). The focus of this study, however, is parties’ emotional experience. According to Jones (2006) it is the *experience* of parties’ emotions that mediators should identify; otherwise, they might miss essential information about the conflict. One important task of a mediator is to help parties focus on the underlying interests signaled by the experience of emotions (Fisher et al., 2011). Furthermore, it is the *experience* of negative emotions that should be addressed, primarily because they can hamper rational thinking (Clore and Huntsinger, 2007; Huntsinger et al., 2014), which could stand in the way of a satisfying mediation agreement (Jones and Bodtker, 2001).

In summary, four questions guide the current study: (a) Do supervisors and subordinates experience emotions differently during mediation? (b) How accurately do mediators perceive emotions experienced by the conflict parties? (c) Is there a difference in the quality of mediators’ perception of subordinate and supervisor emotions? (d) Does the perceptional accuracy differ for positive and negative emotions?

## The Importance of Emotion Recognition in Mediation

Emotions are “episodic, relatively short-term, biologically based patterns of perception, experience, physiology, action, and communication that occur in response to specific physical and social challenges and opportunities” (Keltner and Gross, 1999, p. 468). Emotions prepare people for action when they are threatened, in the case of negative emotions, or when they see opportunities, in the case of positive emotions (Frijda, 1986; Niedenthal and Ric, 2017; Williams and Hinshaw, 2018; Revord et al., 2021). Mediators often observe many different kinds of negative emotions. This may be fear of seeing the other conflict party (“I have to see my manager at the mediation table? Last time I saw her, she was shouting at me!”), anger (“He called in sick when he knew that our company was in trouble!”), or sadness (“Why did he fire me? I thought I meant more to him.”).

Mediation scholars have been asking whether addressing negative emotions is necessary during mediation (Jones and Bodtker, 2001). On the one hand, it can be argued that negative emotions should be put aside because they can complicate the mediation process and result in mediators losing control of the behavior of disputants (Kelly and Kaminskienë, 2016). On the other hand, emotions signal what really matters to parties (Goldberg and Shaw, 2007; Fisher et al., 2011; Kals et al., 2016). Recent research on mediation effectiveness supports the latter perspective, indicating that in hierarchical labor conflicts, mediators should acknowledge negative emotions in order to achieve positive outcomes (Bollen and Euwema, 2014). The opportunity to express negative emotions in a safe setting is an element of giving voice, which contributes to perceptions of fairness (Lind and Tyler, 1988; Shapiro et al., 1994; Judge et al., 2006) and makes people feel listened to and understood (Tjosvold, 1984; Bruneau and Saxe, 2012; Oishi et al., 2013; Gramling et al., 2016). This might be especially important for subordinates who feel less heard and less acknowledged at work, as compared to supervisors (Fitness, 2000).

In addition, a mediator should attend to negative emotions because, if not addressed properly, they can hinder the mediation process. Parties experiencing strong negative emotions can feel overwhelmed, sometimes leading to emotional “flooding” (Bodtker and Katz Jameson, 2001; Nair, 2008) hampering their rational, cognitive functioning (Jones and Bodtker, 2001; Clore and Huntsinger, 2007; Huntsinger et al., 2014). Feeling overwhelmed interferes with a person’s ability to listen to another party and to engage in problem-solving behaviors (Druckman and Olekalns, 2008).

However, the emotions displayed during mediation are not only negative. Parties might also show positive emotions, such as happiness (“I am happy that we are finally talking to each other”) or enthusiasm (“I feel excited because I see a lot of new opportunities”). Mediators should acknowledge and encourage these emotions since they can foster cooperation and facilitate deal-making (Grandey, 2000; Fisher and Shapiro, 2005; Carnevale, 2008). The broaden-and-build theory of positive emotions (Fredrickson, 2001; Johnson and Fredrickson, 2005; Fredrickson and Joiner, 2018) states that positive emotions help people to form new skills over time, broadening their awareness and encouraging novel, varied, and exploratory thoughts and actions. Positive emotions are positively related to creativity and positive coping (Diener et al., 2020). Negotiation research shows that positive emotions are important, as they create a positive climate in which parties are more willing to listen to one another and to come to an agreement (Fisher and Shapiro, 2005). More specifically, they push parties to be more future-focused, to invest more in the relationship, and to perceive their counterpart as more favorable, which may even generate positive emotions in the other party (Barsade, 2002; Kopelman et al., 2006; Olekalns and Druckman, 2014). As mediation can be seen as a guided negotiation by a third party, a mediator should create an environment that offers room for positive emotions in order to work with them.

## Hierarchical Position and Emotion Experience During Mediation

Supervisors and subordinates often begin the mediation on different footings because of their different formal position (Bollen et al., 2010, 2012). Supervisors are usually more powerful than their subordinates (Euwema, 1992) and more able to inflict costs or withhold benefits (Keltner et al., 2003; Galinsky et al., 2011). Because subordinates have more to lose, they are usually more severely affected by the conflict (Eatough and Chang, 2018). Consequently, they often feel uncertain and vulnerable when entering mediation (Bollen et al., 2010).

As numerous researchers point out, power exerts a strong influence on people’s feelings, thoughts, and actions in general (for a review, see Guinote, 2017), especially in times of conflict (Fitness, 2000; Aquino et al., 2006). When in conflict with low-power parties, high-power parties (usually the supervisor, in a hierarchical conflict) tend to behave in a domineering manner, whereas low-power parties’ actions are restricted (Van de Vliert et al., 1995; Coleman and Ferguson, 2014). This can be explained by the approach/inhibition theory of power (Anderson and Berdahl, 2002; Keltner et al., 2003; Cho and Keltner, 2020). This theory posits that powerful individuals are approach-motivated, focusing more on reward, using more automatic cognition, and being more likely to behave in an unconstrained manner. In contrast, people who are lower in power and more inhibition motivated, tend to perceive situations as more threatening, use more controlled cognition, and act with more social constraint. This theory predicts how power influences one’s emotional life. More specifically, it states that people with power are more likely to experience positive emotions, such as happiness and pride, while people without power are more likely to experience negative emotions, such as fear or sadness (Keltner et al., 2003; Berdahl and Martorana, 2006; Langner and Keltner, 2008; Mast and Palese, 2019). Previous research confirms this (Berdahl and Martorana, 2006; Bombari et al., 2017; Van Kleef and Lange, 2020). Thus, our first hypothesis is:

*Hypothesis 1*: (a) Subordinates experience a higher level of negative emotions during the mediation than supervisors do, and (b) supervisors experience a higher level of positive emotions during mediation than subordinates do.

## Accuracy of Emotion Recognition in Hierarchical Workplace Mediations

Following the appraisal theory of emotions (Lazarus, 1991; Burleson and Goldsmith, 1998), which states that the source of emotional distress lies in how events are appraised, not the events themselves, Jones (2006) suggests that mediators need three skills in order to work with emotions: (a) to recognize the emotional experience of a disputant; (b) to help the disputant to understand their own emotional experiences; and (c) to help the disputant to reappraise the emotion by reframing the problem. In this paper, we focus on the first skill. A first crucial step toward recognizing emotions is the analysis of expressive cues, such as facial expressions or body posture, which happens in a quick and automatic manner (Neumann and Strack, 2000; Elfenbein, 2007). For this, the mediator must consider the larger social context (Barrett et al., 2007; Barrett et al., 2011; Hess et al., 2016). Furthermore, the interpretation of emotions is influenced by the perceiver’s knowledge of the situation, culture, social norms, and display rules (Markus and Kitayama, 1991; Kayyal et al., 2015). Consequently, emotion recognition depends also on the perceiver’s interpretive lens (Elfenbein, 2007). All this implies that accurately perceiving conflict parties’ experienced emotions is challenging, especially when mixed- or low-intensity emotions are involved (Scherer and Ceschi, 2000; Thompson et al., 2001; Elfenbein and Ambady, 2002), or when they strategically fake, moderate, or mask emotions (Clark et al., 1996; Wong et al., 2013). In hierarchical labor conflicts, accurately recognizing the experience of emotions may be even more challenging since subordinates and supervisors differ in their emotional expressions (cf. the approach/inhibition theory of power). In other words, subordinates are less likely to show their emotions than supervisors are; thus, recognizing subordinates’ emotions may be more difficult for mediators. Consequently, our second hypothesis is as follows:

*Hypothesis 2*: Mediators in hierarchical workplace conflicts perceive emotions of supervisors more accurately than they do those of subordinates.

Accordingly, we suggest that mediators more accurately perceive parties’ negative emotions than they do their positive emotions. From an evolutionary perspective, people are more sensitive to negative than positive emotions because such emotions imply a direct threat and thus require an immediate response (Frijda, 1986; Cosmides and Tooby, 2000; Izard, 2013). In addition, numerous studies have provided evidence of a “negativity bias.” People in general give more attention and weight to negative stimuli than to positive stimuli (Carretié et al., 2001; Rozin and Royzman, 2001; Ito and Cacioppo, 2005; Bebbington et al., 2017). Consequently, we propose that mediators are more attuned to the experience of negative emotions than to that of positive emotions. For this reason, our third hypothesis is:

*Hypothesis* 3: Mediators in hierarchical workplace conflicts perceive more accurately the experience of parties’ negative emotions than that of their positive emotions.

## Materials and Methods

### Data Collection and Respondents

Prior to conducting this study, a supervisory committee reviewed the research for ethical considerations. Subsequently, we contacted the Dutch Mediation Federation (MfN)^1^. We agreed with the MfN that (a) all data would be processed anonymously, (b) we would guarantee the confidentiality of all information obtained (for example names and email addresses), (c) participation in the research would be voluntary, (d) our research would meet the requirements of scientific research, and (e) the research would result in a scientific paper. The MfN, which places great value on the confidentiality of mediations, gave their permission to carry out the study and provided us with the opportunity to contact certified mediators and their clients. Those mediators who agreed to work with us asked supervisors and subordinates in five of their successive mediations whether they would want to participate in our research. If so, mediators asked them whether they could send parties’ contact information to the researchers. Once we received the contact details, people were informed by email that our research met the requirements of scientific research. Participation was voluntary, and we guaranteed confidentiality: for example, their mediator would not have access to or be informed about their completed questionnaire. We also informed them that the research would result in a scientific paper. Both mediator and parties received a digital questionnaire by email immediately after the final session (within a maximum of 4 weeks), between January 2011 and July 2014. The questionnaire included several measures to assess general information about the conflict and the mediation, including the experience of positive and negative emotions. The mediator also completed a questionnaire on the same topics, assessing supervisors’ and subordinates’ emotions. To avoid selection bias on the part of the mediators, all parties in successive mediations were asked to participate. We also allowed a maximum of five mediations per mediator in our data set to prevent a sample bias.

### Sample

In total, 168 parties and their mediators stemming from 84 mediations received questionnaires. Of these, 41 supervisors (28 male and 13 female), 55 subordinates (24 male and 31 female) and 29 mediators (16 male and 13 female) returned the surveys, yielding a 57% (mediation clients) and 85% (mediators) response rate. In total, we received data from 67 mediations. Most mediators filled out a survey for one or two mediations (83%), while a smaller percentage of mediators sent us data from three or more mediations (17%). In 14% of the cases, we could not link the data of a supervisor or a subordinate to the data of a specific mediator because the mediator did not complete the questionnaire. Of the 96 returned questionnaires (completed by subordinates or supervisors), 58 participants were involved in the same mediation (a total of 29 dyads), while 38 participants were not involved in the same mediation (either a supervisor or subordinate returned the survey). This implies that in most cases (60%), both supervisors and subordinates were involved in the same mediation. On average, supervisors were aged 47.05 years (*SD* = 7.18), and subordinates 49.55 years (*SD* = 9.22). Approximately 88% of the supervisors had a degree in higher education (*N* = 36), of which 42% had a university degree. Some 68% of the subordinates had a higher educational degree (*N* = 37), of which 20% had a university degree. The average age of the mediators was 52.93 years (*SD* = 8.00). The mediators all had a higher educational degree, of which 69% (*N* = 20) had a university degree. They were experienced, with 83% (*N* = 24) having mediated for more than 5 years and with 83% (*N* = 24) conducting more than 20 mediations per year, 14% (*N* = 4) conducting 10–20 per year and 3% (*N* = 1) conducting 5–10 per year. All respondents (mediators and clients) were Dutch. The data show that the conflicts tended to be highly escalated as perceived by mediation clients, with an average escalation level of 4.03 (*SD* = 1.08) on a 5-point Likert scale, ranging from 1 to 5, reflecting a very high level of escalation. In approximately 80% of all cases, the parties reached an agreement. This is in line with earlier research indicating that agreement rates for (workplace) mediation vary from 60 to 80% (McDermott et al., 2002; Poitras and Le Tareau, 2009).

### Measures

*Hierarchical position.* In this study, hierarchical position is operationalized as the position of authority or certain formal position in relation to the other party in the mediation (Finkelstein, 1992) (“What is your position in the conflict?”). The possible categories were as follows: employer, employee, and other. Employee refers to subordinates and employer to supervisors. Subordinates were coded as 1, and supervisors, as 2.

*Mediation clients’ emotions.* We created a Positive Emotions Scale and a Negative Emotions Scale based on the existing Positive and Negative Affect Schedule (PANAS) developed by Watson et al. (1988) to measure the emotions of mediation clients. To measure positive emotions, we asked the parties to indicate the extent to which they felt happy and enthusiastic during the mediation (two items) (*r* = 0.69) (“To what extent did you feel happy during the mediation?”*).* To measure negative emotions, we asked the parties to indicate the extent to which they experienced anger, fear, and sadness (three items) (α = 0.78) (“To what extent did you feel angry during the mediation?”). We coded the responses to the different items on a 5-point Likert scale (1 = *not at all*; 5 = *extremely*), that indicated whether they were experiencing specific emotions.

*Perception of parties’ emotions by mediators.* Mediators received surveys similar to those of the clients, asking them to indicate the extent to which they perceived the positive (two items) and negative (three items) emotions of subordinates and supervisors. The survey asked them to measure the supervisors’ levels of happiness and enthusiasm during mediation (two items) (“The supervisor was happy during the mediation”) (*r* = 0.83), and those of the subordinates (*r* = 0.72). The survey also asked respondents to indicate the levels of anger, fear and sadness experienced by supervisors (three items α = 0.80) (“The supervisor was angry during the mediation”) and subordinates (α = 0.58) on a 5-point Likert scale.

### Data Analyses

Data management and analyses were executed using SPSS 24.0. We used MANCOVA analyses and hierarchical regression analyses to test our hypotheses. As the experience of emotions may depend on the gender of the clients (1 = *male*, 2 = *female*), the escalation level of the initial conflict as perceived by the party (on a 5-point Likert scale: 1 = *not at all*, 5 = *to a great extent*) and mediation outcome (0 = *no agreement*, 1 = *agreement*), we controlled for these variables (Bollen and Euwema, 2014).

## Results

**FIGURE 1:**
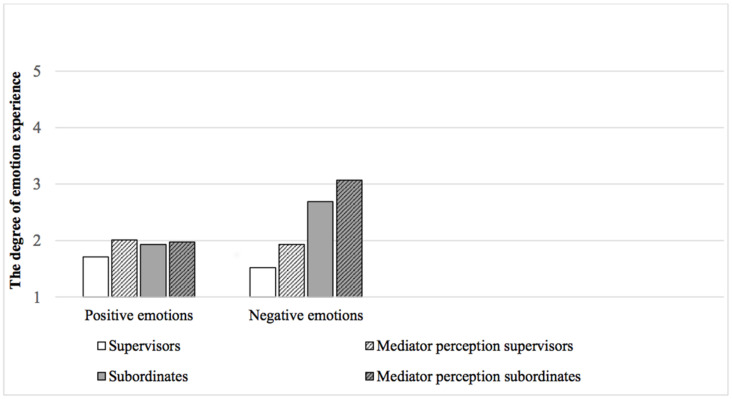
Hierarchical position, experience of emotion, and mediators’ perceptions of emotion.

Table 1 presents mean scores, standard deviations, and correlations among control and research variables. Figure 1 provides the results for hierarchical position, experience of emotion, and mediators’ perceptions of emotion.

**TABLE 1:** Means (M), standard deviations (SD), and correlations between parties’ positive emotions, negative emotions, mediator emotion perception and control variables.

	**M**	**SD**	**1**	**2**	**3**	**4**	**5**	**6**	**7**	**8**	**9**	**10**
1. Gender clients (*N* = 96)	1.46	0.50										
2. Conflict escalation (*N* = 96)	4.03	1.08	0.11									
3. Agreement (*N* = 96)	0.82	0.39	0.15	0.02								
4. Positive emotions subordinates (*N* = 55)	1.93	1.13	–0.06	0.10	–0.05							
5. Negative emotions subordinates (*N* = 55)	2.69	1.10	0.11	0.29*	0.09	–0.27						
6. Mediator perception subordinates’ positive emotions (*N* = 49)	1.97	1.08	–0.11	0.33*	0.29	–0.01	–0.12					
7. Mediator perception subordinates’ negative emotions (*N* = 49)	3.07	0.96	0.08	0.10	0.11	0.10	0.09	0.06				
8. Positive emotions supervisors (*N* = 41)	1.71	0.93	–0.04	–0.21	0.21	–	–	–	–			
9. Negative emotions supervisors (*N* = 41)	1.52	0.61	0.13	0.35*	–0.09	–	–	–	–	–0.17		
10. Mediator perception supervisors’ positive emotions (*N* = 37)	2.01	1.25	0.02	0.26	0.15	–	–	–	–	0.40*	0.06	
11. Mediator perception supervisors’ negative emotions (*N* = 37)	1.93	1.05	–0.04	0.55**	−0.51**	–	–	–	–	–0.08	0.59**	0.33

*p ≤ 0.05, **p ≤ 0.01.

Hypothesis 1 states that (a) subordinates experience a higher level of negative emotions during the mediation than supervisors do, and that (b) supervisors experience a higher level of positive emotions during the mediation than subordinates do. The results indicate that, as expected, hierarchical position is significantly related to the experience of negative emotions during mediation (*r* = −0.54, *p* < 0.001). However, this does not apply to the experience of positive emotions (*r* = −0.11, *p* > 0.05), as is confirmed by a MANCOVA analysis of negative emotions [*F*_(1,82)_ = 23.72, *p* < 0.001] and positive emotions [*F*_(1,82)_ = 1.14, *p* > 0.05]. Subordinates experience a significantly higher level of negative emotions during mediation than supervisors do (*M* = 2.69, *SD* = 1.10 vs. *M* = 1.52, *SD* = 0.61), including when controlling for the clients’ gender [*F*_(1,82)_ = 2.59, *p* > 0.05], conflict escalation [*F*_(1,82)_ = 7.07, *p* < 0.01] and mediation outcome (agreement or not) [*F*_(1,82)_ = 0.34, *p* > 0.05]. However, supervisors do not experience a higher level of positive emotions during mediation than subordinates do (*M* = 1.71, *SD* = 0.93 vs. *M* = 1.93, *SD* = 1.13). We found the same result when we only tested the matching data (supervisors and subordinates who were in the same mediation). Here, subordinates also significantly experience a higher level of negative emotions than supervisors do (*M* = 2.41, *SD* = 0.93 vs. *M* = 1.53, *SD* = 0.55), including when controlling for the clients’ gender [*F*_(1,48)_ = 2.93, *p* > 0.05], conflict escalation [*F*_(1,48)_ = 4.26, *p* < 0.05] and mediation outcome (agreement or not) [*F*_(1,48)_ = 0.38, *p* > 0.05]. Subordinates and supervisors experience the same level of positive emotions (*M* = 1.92, *SD* = 1.12 vs. *M* = 1.54, *SD* = 0.73). Thus, Hypothesis 1 is partly confirmed for (a) but not for (b).

**FIGURE 2:**
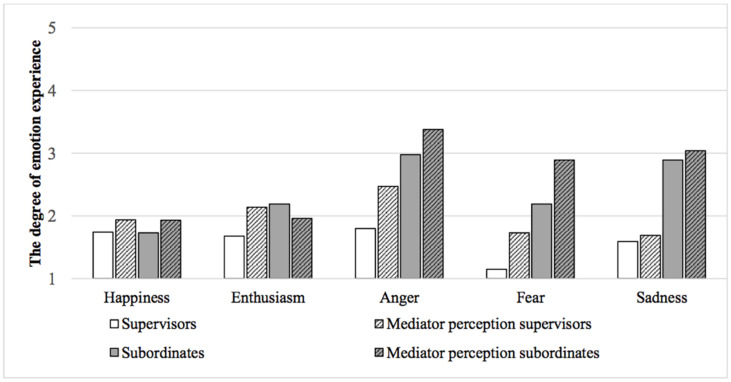
Hierarchical position, experience of independent emotions and mediators’ perceptions of independent emotions.

In Table 2 (supervisors) and Table 3 (subordinates), means, standard deviations and intercorrelations among research variables are displayed for independent emotions. Figure 2 provides the results for hierarchical position, experience of independent emotions and mediators’ perceptions of independent emotions.

**TABLE 2:** Means (M), standard deviations (SD), and correlations between supervisors’ emotions happiness, enthusiasm, anger, fear, sadness and mediator emotion perception.

	***M***	***SD***	**1**	**2**	**3**	**4**	**5**	**6**	**7**	**8**	**9**
1. Happiness supervisor (*N* = *41)*	1.74	0.97									
2. Enthusiasm supervisor (*N* = *41)*	1.68	1.05	0.69**								
3. Anger supervisor (*N* = *41)*	1.80	1.04	–0.07	–0.16							
4. Fear supervisor (*N* = *41)*	1.15	0.53	–0.18	–0.19	0.29						
5. Sadness supervisor (*N* = *41)*	1.59	0.94	0.11	–0.22	0.22	0.34*					
6. M. perception happiness (*N* = *37)*	1.94	1.39	0.39*	0.25	0.00	0.00	0.07				
7. M. perception enthusiasm (*N* = *37)*	2.14	1.22	0.39*	0.25	0.10	0.07	0.11	0.83**			
8. M. perception anger (*N* = *37)*	2.47	1.25	–0.24	–0.24	0.40*	0.45**	0.24	0.11	0.20		
9. M. perception fear (*N* = *37)*	1.73	1.24	0.05	–0.05	0.28	0.47**	0.42*	0.47**	0.37*	0.54**	
10. M. perception sadness (*N* = *37)*	1.69	1.26	0.15	0.07	0.22	0.59**	0.47**	0.25	0.16	0.50**	0.66**

*p ≤ 0.05, **p ≤ 0.01.

**TABLE 3:** Means (M), standard deviations (SD), and correlations between subordinates’ emotions happiness, enthusiasm, anger, fear, sadness and mediator emotion perception.

	***M***	***SD***	**1**	**2**	**3**	**4**	**5**	**6**	**7**	**8**	**9**
1. Happiness subordinate (*N* = 55)	1.73	1.12									
2. Enthusiasm subordinate (*N* = 55)	2.19	1.32	0.71**								
3. Anger subordinate (*N* = 55)	2.98	1.34	–0.25	–0.27							
4. Fear subordinate (*N* = 55)	2.19	1.32	–0.11	–0.04	0.42**						
5. Sadness subordinate (*N* = 55)	2.89	1.41	–0.24	−0.29*	0.45**	0.56**					
6. M. perception happiness (*N* = 49)	1.93	1.20	–0.13	0.01	–0.06	–0.15	–0.21				
7. M. perception enthusiasm (*N* = 49)	1.96	1.13	0.07	0.26	0.09	–0.11	–0.14	0.72**			
8. M. perception anger (*N* = 49)	3.38	1.13	0.07	–0.03	0.21	–0.14	–0.12	0.03	0.32*		
9. M. perception fear (*N* = 49)	2.89	1.35	0.09	0.12	0.06	0.05	–0.09	–0.07	–0.02	0.30*	
10. M. perception sadness (*N* = 49)	3.04	1.30	0.03	0.09	0.11	0.22	0.29	–0.01	0.15	0.23	0.41**

*p ≤ 0.05, **p ≤ 0.01 1. The MfN is the Dutch national standard-setting and quality assurance platform for the mediation practice in the Netherlands.

Testing of the relationship between hierarchical position and the independent emotions reveals that the negative emotions and the positive emotion of enthusiasm are significantly related to hierarchical position: anger (*r* = −0.44, *p* < 0.001), fear (*r* = −0.47, *p* < 0.001), sadness (*r* = −0.47, *p* < 0.001), and enthusiasm (*r* = −0.20, *p* < 0.05). No significant relationship exists between hierarchical position and the experience of happiness (*r* = 0.01, *p* > 0.05). MANCOVA analysis confirms these results for the negative emotions of anger [*F*_(1,82)_ = 13.73, *p* < 0.001], fear [*F*_(1,82)_ = 12.70, *p* = 0.001], and sadness [*F*_(1,82)_ = 14.28, *p* < 0.001], but not for the positive emotion of enthusiasm [*F*_(1,82)_ = 3.36, *p* > 0.05]. Subordinates experience significantly higher levels of negative emotions than supervisors do: anger (*M* = 2.98, *SD* = 1.34 vs. *M* = 1.80, *SD* = 1.04), fear (*M* = 2.19, *SD* = 1.32 vs. *M* = 1.15, *SD* = 0.53), and sadness (*M* = 2.89, *SD* = 1.41 vs. *M* = 1.59, *SD* = 0.94), even when controlling for the clients’ gender, conflict escalation and mediation outcome (agreement or not). Subordinates and supervisors do not experience significantly different levels of positive emotions: happiness (*M* = 1.73, *SD* = 1.12 vs. *M* = 1.74, *SD* = 0.97) and enthusiasm (*M* = 2.19, *SD* = 1.32 vs. *M* = 1.68, *SD* = 1.05). When testing the matched data (only data that included supervisors and subordinates in the same mediation), we found largely the same result as in the whole sample for the negative emotions of anger [*F*_(1,48)_ = 4.38, *p* < 0.05], fear [*F*_(1,48)_ = 9.50, *p* < 0.01], and sadness [*F*_(1,48)_ = 6.15, *p* < 0.05]. Subordinates experience significantly higher levels of negative emotions than supervisors do: anger (*M* = 2.69, *SD* = 1.32 vs. *M* = 1.85, *SD* = 1.06), fear (*M* = 1.92, *SD* = 1.13 vs. *M* = 1.11, *SD* = 0.32), and sadness (*M* = 2.62, *SD* = 1.42 vs. *M* = 1.63, *SD* = 1.01), even when controlling for the clients’ gender, conflict escalation and mediation outcome (agreement or not). Furthermore, subordinates and supervisors do not experience significantly different levels of happiness (*M* = 1.73, *SD* = 1.15 vs. *M* = 1.63, *SD* = 0.84). However, contrary to the whole sample, subordinates in matching dyads experience significantly more enthusiasm during mediation than supervisors do [*F*_(1,48)_ = 4.23, *p* < 0.05; *M* = 2.12, *SD* = 1.37 vs. *M* = 1.44, *SD* = 0.89].

Hypothesis 2 states that mediators in hierarchical workplace conflicts perceive the positive and negative emotions of supervisors more accurately than they do those of subordinates. The results indicate, as expected, that the positive emotions reported by supervisors are positively related to mediator recognition of these emotions (*r* = 0.40, *p* < 0.05) (see Table 1). This also applies to negative emotions (*r* = 0.59, *p* < 0.01). Hierarchical regression analyses confirm these findings for positive emotions (β = 0.40, *p* < 0.05), including when controlling for gender of the clients (β = 0.03, *p* > 0.05), conflict escalation level (β = 0.30, *p* > 0.05) and mediation outcome (agreement or not) (β = 0.18, *p* > 0.05). We also see that negative emotions self-reported by supervisors are positively related to mediators’ recognition of these emotions (β = 0.59, *p* < 0.001). This finding holds when controlling for gender of the clients (β = −0.11, *p* > 0.05), escalation level (β = 0.37, *p* < 0.01) and mediation outcome (agreement or not) (β = −0.40, *p* = 0.001). The results show no significant relationship between the positive emotions reported by subordinates and mediator recognition of these emotions (*r* = −0.01, *p* > 0.05) (see Table 1). This also applies to negative emotions (*r* = 0.09, *p* > 0.05). Hierarchical regression analyses confirm these findings for both positive emotions (β = −0.01, *p* > 0.05) and negative emotions (β = 0.09, *p* > 0.05). Thus, Hypothesis 2 is supported. When we consider the emotions as experienced independently by supervisors and as perceived by mediators (Table 2), we see that four of the five emotions are positively related to mediators’ recognition of them: happiness (*r* = 0.39, p < 0.05), anger (*r* = 0.40, *p* < 0.05), fear (*r* = 0.47, *p* < 0.01), and sadness (*r* = 0.47, *p* < 0.01). There is no significant relationship between supervisors’ enthusiasm and mediators’ perception of this enthusiasm (*r* = 0.25, *p* > 0.05). Hierarchical regression analyses show that supervisors’ experience of happiness (β = 0.39, *p* < 0.05), fear (β = 0.47, *p* < 0.01), and sadness (β = 0.47, *p* < 0.01) are related to mediators’ perceptions of these emotions. There is no significant relationship however between supervisors’ anger and mediators’ anger perception (β = 0.21, *p* > 0.05) after controlling for gender, escalation level and agreement. Similarly, no relationship was found between supervisors’ enthusiasm and mediators’ perception of this enthusiasm (β = 0.25, *p* > 0.05). With respect to the emotions as experienced independently by subordinates and in terms of their perception by mediators (Table 3), we see that none of the five emotions are related to mediators’ perceptions of that particular emotion: happiness (*r* = −0.13, *p* > 0.05), enthusiasm (*r* = 0.26, *p* > 0.05), anger (*r* = 0.21, *p* > 0.05), fear (*r* = 0.05, *p* > 0.05), and sadness (*r* = 0.29, *p* > 0.05). Hierarchical regression analyses confirm that no relationship exists between the emotions as experienced by subordinates and the perceptions of these emotions by mediators: happiness (β = −0.13, *p* > 0.05), enthusiasm (β = 0.26, *p* > 0.05), anger (β = 0.21, *p* > 0.05), fear (β = 0.05, *p* > 0.05), and sadness (β = 0.29, *p* > 0.05).

Hypothesis 3 states that mediators in hierarchical workplace conflicts perceive negative emotions more accurately than positive emotions. Our findings show that, as expected, mediators perceive negative emotions of supervisors (*r* = 0.59, *p* < 0.01) more accurately than they do positive emotions (*r* = 0.40, *p* < 0.05). We find no significant relation between mediators’ perception and self-reports of subordinates’ negative emotions (*r* = 0.09, *p* > 0.05) or positive emotions (*r* = −0.01, *p* > 0.05).

## Discussion

Mediation is widely used to constructively resolve workplace conflicts, including hierarchical workplace conflict (Bollen and Euwema, 2013; Munduate et al., 2016; Kalter et al., 2018). In this study, we examined whether supervisors and subordinates differ in their emotional experience during mediation, the extent to which mediators can correctly perceive positive and negative emotions and whether they perceive negative emotions more efficiently than positive emotions.

Our study reveals that parties who occupy different hierarchical positions have a different emotional experience during mediation. More specifically, subordinates experience higher levels of negative emotions, such as anger, fear and sadness, than supervisors do. These outcomes are in line with the approach/inhibition theory of power (Anderson and Berdahl, 2002; Keltner et al., 2003; Cho and Keltner, 2020). In contrast to expectations, supervisors and subordinates experienced the same levels of positive emotions during mediation. A possible explanation might be that mediation is about resolving disputes (Moore, 2014). If both supervisor and subordinate feel that a resolution of the problem is at hand, they are likely to experience positive emotions as a result. In addition, subordinates may feel empowered by the mediation process (Bush and Folger, 2004), resulting in positive emotions.

Moreover, our study indicates that mediators more accurately perceive the emotions of supervisors than those of subordinates. This difference holds for both positive and negative emotions. While mediator perceptions of supervisors’ happiness, sadness and fear are aligned with the supervisors’ own experiences, there is no significant relationship between mediator perceptions and subordinates’ emotions. These findings indicate that hierarchy continues to play a role in workplace mediations. Not only do supervisors and subordinates experience the mediation and its effects differently (e.g., Coggburn et al., 2018; Kalter et al., 2018; Kalter, 2020), mediators are also indirectly affected by the hierarchical position parties occupy. Mediators lower accuracy in perceiving subordinates’ emotions could be due to subordinates’ caution in showing their emotions, as predicted by the approach/inhibition theory of power (Keltner et al., 2003). Strikingly, mediators generally estimate parties’ emotions to be higher on a scale from 1 to 5 than parties do themselves. Possibly, the self-report measures of parties’ emotion experiences may have resulted in minimizing actual emotions because parties wanted to present themselves favorably (Donaldson and Grant-Vallone, 2002) or because mediators perceive the mediation as more of an emotional process than parties do and therefore interpret emotional cues as more “intense” (Elfenbein, 2007).

Finally, our study shows that mediators more accurately perceive negative emotions than positive emotions of supervisors. This emphasis on the negative may reflect the fact that people more often attend to negative stimuli than to positive stimuli (Carretié et al., 2001), indicating a “negativity bias” (Rozin and Royzman, 2001; Huang and Luo, 2006).

### Strengths, Limitations, and Avenues for Future Research

The present study variously contributes to the research on mediation. First, previous studies have mainly focused on the importance of addressing emotions (Jones, 2006; Picard and Siltanen, 2013; Bollen and Euwema, 2014). However, this study considerably extends that focus by investigating parties’ emotional experiences during mediation and mediator perceptions of these emotions.

Second, in contrast to previous research that concentrates on anger (Jones and Bodtker, 2001; Bollen and Euwema, 2014), our study also considers other negative emotions, such as fear and sadness, that are important for mediators to acknowledge. Furthermore, the positive emotions of happiness and enthusiasm were part of our research, since they play an important role in mediation by fostering cooperation and deal-making (Grandey, 2000; Fisher and Shapiro, 2005; Kopelman et al., 2006; Carnevale, 2008; Olekalns and Druckman, 2014).

Third, although there have been studies on mediator perception (e.g., Mareschal, 2005; Swaab and Brett, 2007), this is the first study involving mediators and at least one of the parties in a mediation. This allowed us to examine the degree of similarity between mediators’ perceptions of parties’ emotions and parties’ emotion experiences.

In addition, there are some reasons to exercise caution when interpreting the findings of this study. Our use of self-report measures raises concerns about socially desirable answers (Paulhus, 1991; Kuncel and Tellegen, 2009). Supervisors and subordinates may have presented themselves in a favorable light. Studies on organizational display rules show that maintaining professionalism is central to appropriate emotion management (Flam, 2002; Kramer and Hess, 2002). For example, supervisors may have minimized their experience of emotions that did not align with the display rules for their hierarchical position, such as fear or sadness. In this respect, selection bias may also have occurred (Collier et al., 2004). Subordinates who experienced strong emotions may have wanted to participate in our study in order to vent. Conversely, some supervisors who felt strong emotions may have been embarrassed and opted out of participation.

Furthermore, we asked mediation clients about their emotion experienced in retrospect. Although we contacted them immediately (within 4 weeks) after the last mediation session to assess their emotions during the mediation process, the whole mediation could have taken place over several months. As such, there may have been a considerable time lag between the experience and assessment of emotions, resulting in a recall bias. It is therefore possible that participants’ responses were affected by their beliefs regarding how they should have felt, rather than how they actually did feel (Robinson and Clore, 2002; Barclay et al., 2005). To overcome these methodological limitations, researchers should include (quasi-) experimental studies and observational research.

Another limitation is that we used a single item measure of discrete emotions based on the Positive and Negative Affect Schedule (PANAS) (Watson et al., 1988). Although many researchers have used the PANAS or have used single items to measure experienced emotions (e.g., Gross and Levenson, 1993), and this approach has been effective, single-item measures are often considered as methodologically suspect (Wanous and Hudy, 2001). For example, they are considered to have lower content validity and to lack a measure of internal-consistency reliability (Rubio et al., 2003). In our study, the use of a single item to measure a certain emotion may not have correctly captured the participant’s subjective experience of this emotion. Future research could use multi-item scales to measure a discrete emotion. For example, Harmon-Jones et al. (2016) have developed a new validated instrument (the DEQ) for measuring eight discrete emotions—anger, disgust, fear, anxiety, sadness, desire, relaxation and happiness—consisting of 32 items (four items per discrete emotion), which could be very useful in this type of research.

Furthermore, we have considered only disputants’ emotional experience, and not their emotional expression, which may or may not correspond (Gross and Levenson, 1993; Davis, 1995; Tamir et al., 2008; Gibson and Callister, 2010). For example, we did not measure the extent to which supervisors and subordinates seek to hide their emotions, while this behavior seems relevant, in light of the assumption that subordinates are more likely to hide their emotions than supervisors do (Fitness, 2000). Discrepancies between felt and expressed emotions may have a negative effect on the mediation. Past research shows that faking emotions has negative consequences for one’s authenticity (Côteí, 2005) and credibility (Andrade and Ho, 2009). Studies of negotiation, for instance, find that faking anger creates mistrust and reduces cooperative behavior from counterparts (Côteí et al., 2013; Campagna et al., 2016). Furthermore, masking emotions can have detrimental effects, potentially leading to conflict escalation and dysfunctional behaviors (Vuori et al., 2018). Future research could explore whether there is a discrepancy between felt and expressed emotions and how this discrepancy affects mediation. Relatedly, the question remains whether mediators are able to detect faked or masked emotions.

This study is the first of its kind, focusing on the extent to which mediators perceive supervisors’ and subordinates’ emotions, including both positive and negative emotions. For future research, it would be interesting to run dyadic analyses. Such analysis is often challenging, given the amount of data needed and the fact people do not share mediation data easily. When we analyzed the matching data in our dataset (a limited number of 29 sets of supervisors and subordinates who were in the same mediation), we found that subordinates felt significantly more enthusiastic than supervisors, but we did not find this result across the whole sample. Dyadic analysis could shed more light on this result. Dyadic analyses could enable researchers to examine the interpersonal effects of emotions in mediation (Fischer and Van Kleef, 2010; Van Kleef, 2016). For example, a question remains as to how supervisors and subordinates trigger emotions in one another during mediation and how this interplay hinders or helps the mediation process.

Another area for future research would be to examine the role of emotional intelligence in effectively mediating disputing parties (Goleman, 2006; Kelly and Kaminskienë, 2016; Valente and Lourenço, 2020). Emotional intelligence is “the ability to perceive emotions, to access and generate emotions so as to assist thought, to understand emotions and emotional knowledge, and to reflectively regulate emotions so as to promote emotional and intellectual growth” (Mayer and Salovey, 1997, p. 10). This study focused on accurate perception of emotion, which is only one aspect of emotional intelligence that may determine mediation effectiveness. Other aspects include knowledge of emotion and the ability to manage emotions (George, 2000), which may aid mediators with helping disputants to understand their own emotional experiences (Jones, 2006). More research is needed to explore how these different aspects of emotional intelligence may enhance the effectiveness of mediators’ practice.

### Practical Implications

Mediating hierarchical workplace mediations can be challenging. The results of the present study illustrate that in this type of mediation, subordinates experience more intense negative emotions than supervisors do. However, mediators can more accurately perceive the emotions of supervisors. For this reason, our results suggest that mediators should pay special attention to the exploration of subordinates’ emotions. This focal point could be important, since a satisfying mediation agreement is more likely if a mediator addresses underlying emotions (Jones and Bodtker, 2001; Jones, 2006). One way to achieve this is through a pre-caucus before the joint face-to-face session or the implementation of a caucus during the mediation (Swaab and Brett, 2007; Charkoudian et al., 2009; Poitras and Raines, 2013). This might be particularly beneficial to subordinates who feel restrained in showing their emotions (Keltner et al., 2003). A one-to-one meeting may provide them with a safe environment in which to express how they feel (Bollen and Euwema, 2014). Although this cannot be directly concluded from our research, this also suggests that it is important that mediators verify assumptions about perceived emotions since our research shows that they do not accurately perceive subordinates’ emotional experiences. In this respect, they might ask clarification questions, such as “you are still angry, is that correct?” or “how would that make you feel?” to determine whether they are correctly reading emotions (Jones and Bodtker, 2001; Kalff and Uitslag, 2007). Furthermore, although our research did not consider whether a mediation was conducted by one mediator or two, mediators may benefit from a co-mediator being present during the sessions (Love and Stulberg, 1996; Marinova, 2008), as four eyes see more than two. For example, while one mediator asks questions and focuses on the big picture, the other could take notes and observe, paying special attention to non-verbal emotion cues.

## Conclusion

The current research demonstrates that hierarchy affects mediators’ perceptions of emotion. Specifically, mediators can correctly perceive the extent to which both positive and negative emotions are experienced by supervisors, but they cannot do this in the case of subordinates. Our study shows that mediators can better perceive negative emotions than positive ones. Furthermore, parties who occupy different hierarchical positions experience emotions differently during mediation. More specifically, subordinates experience higher levels of negative emotions than supervisors do.

## Data Availability Statement

The raw data supporting the conclusions of this article will be made available by the authors, without undue reservation.

## Ethics Statement

Written informed consent for participation was not required for this study in accordance with the national legislation and the institutional requirements at the time of data collection.

## Author Contributions

MK, ME, and KB contributed to conception and design of the study. MK organized the database and performed the statistical analysis and wrote the first draft of the manuscript. A-LV revised the work critically for important intellectual content. All authors contributed to manuscript revision, read and approved the submitted version.

## Conflict of Interest

The authors declare that the research was conducted in the absence of any commercial or financial relationships that could be construed as a potential conflict of interest.
